# Supplementary material for: Menstrual Cycle-Related Hormonal Fluctuations in ADHD: Effect on Cognitive Functioning—A Narrative Review
Source: J Clin Med. 2025 Dec 24;15(1):121. doi: 10.3390/jcm15010121 (PMC12786913; doi:10.3390/jcm15010121)
Supplement: Supplementary file 1 [file jcm-15-00121-s001.zip › jcm-3963525-supplementary.pdf]

Supplementary Table S1. Summary of findings of studies that examined the effect of the menstrual cycle on cognitive symptoms in ADHD*

Author/year	Subjects	Measured Domains (instrument)	Phase Verification Method	Main results
**Menstrual**				
Bürger et al. (2024) [49]	NCF with ADHD = 10	ADHD symptoms (interviews)	Self-report tracking [b]	↑ADHD symptoms (Executive dysfunction, Emotional dysregulation, Inattention)
Diekhof (2015) [51]	NCF = 28 (low trait impulsivity = 14)	Impulsivity (reward acquisition paradigm)<br>Trait impulsivity (Barrett Impulsiveness Scale)	Self-report tracking [c]<br>Saliva samples	↑Impulsive choices<br>E2 levels correlated positively with impulsive choices during the menstrual phase, particularly in females with low trait impulsiveness
Hidalgo-Lopez & Pletzer (2021) [43]	NCF = 39	WM (n-back test)<br>Executive function (n-back test)	Saliva samples	No menstrual cycle effects observed in behavioural measures<br>Different brain connectivity patterns, depending on menstrual cycle phase<br>↓Connectivity between fronto-striatal areas and regions related to salience and cognitive effort
Ronca et al. (2025) [34]	Males = 96<br>NCF = 105<br>OCF = 47	Sustained attention (Smiley task battery)<br>Inhibition (Spatial simple reaction time)<br>Visuospatial function (Cube Analysis test)<br>Mood (Burgess Brief Mood Questionnaire)	Self-report tracking [b]<br>Backwards counting [b]	↑Cognitive performance, Faster RT, ↓Mood in NCF
Schmalenberger et al. (2024) [46]	NCF = 19 (with suicidal ideation)	WM (n-back)<br>Verbal fluency (Verbal fluency test)<br>Inhibition (Stop-signal task)	E2 transdermal patch<br>Placebo patch<br>Placebo pill	↓WM, ↓Verbal fluency in placebo condition<br>E2 administration (regardless of additional P4) prevented decreased WM and verbal fluency performance during the (pre-)menstrual phase
**Follicular (mid-/early)**				
Eggert et al. (2017) [41]	PMS = 55<br>Non-PMS = 55	Attention, Executive Function (Emotional Stroop task)	Self-report tracking [b]	↑Emotional Stroop effect in non-PMS women
Gaizauskaite et al. (2025) [42]	Males = 32<br>NCF = 133	WM (Bilateral change detection task)	Saliva samples	No systematic differences in WM between groups nor any correlations with hormone levels

	OCF = 37<br>IUD = 28			↓WM with increasing task difficulty
Leeners et al. (2017) [44]	First cycle:<br>NCF = 88,<br>Second cycle:<br>NCF = 68	WM (block span test)<br>Attention (Divided Attention Bimodal task)<br>Cognitive control (Cognitive Bias test)	Blood samples<br>Transvaginal ultrasound	↓Attention in first cycle only<br>No findings replicated during the second menstrual cycle
Roberts et al. (2018) [53]	NCF = 32	ADHD symptoms (Current ADHD Symptoms Scale: Self-report)<br>Trait impulsivity (Urgency, Premeditation, Perseverance, Sensation Seeking-Positive Urgency Trait Impulsivity Scale)	Saliva samples (daily)	↑Hyperactivity/impulsivity, especially with high trait impulsivity
**(Pre- and post-)Ovulatory**				
Leeners et al. (2017) [44]	First cycle:<br>NCF = 88,<br>Second cycle:<br>NCF = 68	WM (block span test)<br>Attention (Divided Attention Bimodal task)<br>Cognitive control (Cognitive Bias test)	Blood samples<br>Transvaginal ultrasound	↑Attention in first cycle only<br>No findings replicated during the second menstrual cycle
Li & Deng (2022) [29]	NCF = 36	Social cognition (visual search task with social and object distractors)<br>Attention (eye-tracking)	Backwards counting [b]	Slower RT, ↓Fixation on social distractors
Lin et al. (2024) [37]	PMDD = 58<br>No PMDD = 50	ADHD symptoms (psychiatric assessment)<br>Attention (Attention and Performance Self-Assessment scale)<br>Impulsivity (Dickman Impulsivity Inventory)	Self-report tracking [c]<br>Ovulation test	Diagnostic criteria for ADHD met significantly more often in PMDD group<br>↓Attention in PMDD, ↑Impulsivity in PMDD (with ADHD)
Pletzer et al. (2019) [52]	NCF = 36	Spatial navigation (landmark test)<br>Verbal fluency (verbal fluency test)	Saliva samples<br>Ovulation test	No significant cognitive performance differences along the menstrual cycle<br>E2 boosts hippocampal activation

Roberts et al. (2018) [53]	NCF = 32	ADHD symptoms (Current ADHD Symptoms Scale: Self-report)<br>Trait impulsivity (UPPS-P Trait Impulsivity Scale)	Saliva samples (daily)	↑Inattention symptoms, ↑Hyperactivity/impulsivity symptoms
Ronca et al. (2025) [34]	Males = 96<br>NCF = 105<br>OCF = 47	Sustained attention (Smiley task battery)<br>Inhibition (Spatial simple reaction time)<br>Visuospatial function (Cube Analysis test)<br>Mood (Burgess Brief Mood Questionnaire)	Self-report tracking [b]<br>Backwards counting [b]	↓Sustained attention in NCF
Yen et al. (2023) [38]	PMDD = 63<br>No PMDD = 53	Inhibition (Go/no-go task)<br>Attention (Go trials in Go/no-go task)<br>Impulsivity (Dickman's Impulsivity Inventory)	Self-report tracking [b]	↓Inhibition, ↑Impulsivity in PMDD
Zhuang et al. (2020) [54]	NCF = 16	Impulsivity (Monetary delay discounting task)<br>Resting-state fMRI<br>Task-based fMRI	Backwards counting [b]	↑Responsivity to short-term rewards, ↑Activity in dorsal striatum, Dorsal striatum-dlPFC connectivity magnitude correlated negatively with impulsivity
**Mid-luteal**				
Alkanat et al. (2021) [30]	NCF = 40	Divided attention (Annett's peg moving task + Go/no-go task)	Ovulation test	↑Divided attention
Blaser et al. (2024) [35]	Low PMS = 36<br>High PMS = 29	Attention (Attentional network test-R with emotional stimuli)	Forward and backwards counting [b]	↓ Attentional control in high PMS
Brotzner et al. (2015) [28]	NCF = 18	Spatial attention (Visuospatial cued attention task)	Saliva samples<br>Ovulation test	↑Attention, RT correlated positively to P4 levels
Bürger et al. (2024) [49]	NCF with ADHD = 10	ADHD symptoms (interviews)	Self-report tracking [b]	↑ADHD symptoms (Executive dysfunction, Emotional dysregulation, Inattention), ↓Effectiveness medication

Cohen et al. (2022) [26]	NCF = 21<br>OCF = 24	Attention (Attentional network test – interactions with alerting and no-alerting condition)	Saliva samples	No significant differences between phases in attention in OCF group<br>↑Alertness mediated by P4 in NCF, ↑Interference in incongruent trials
Hidalgo-Lopez & Pletzer (2017) [39]	NCF = 36	WM (n-back test)<br>Executive function (Stroop task)	Saliva samples	↑WM when ↑P4, ↑Baseline DA was related to ↓Inhibition,<br>↓Eyeblink-rate was related to ↑Inhibition
Hidalgo-Lopez & Pletzer (2021) [43]	NCF = 39	WM (n-back test)<br>Executive function (n-back test)	Saliva samples	↑Connectivity between fronto-striatal areas, posteromedial structures, and regions related to salience and cognitive effort
Leeners et al. (2017) [44]	First cycle: NCF = 88,<br>Second cycle: NCF = 68	WM (block span test)<br>Attention (Divided Attention Bimodal task)<br>Cognitive control (Cognitive Bias test)	Blood samples<br>Transvaginal ultrasound	P4 negatively correlated to WM in first cycle only<br>No findings replicated during the second menstrual cycle
Li & Deng (2022) [29]	NCF = 36	Social cognition (visual search task with social and object distractors)<br>Attention (eye-tracking)	Backwards counting [b]	Faster RT, ↑Fixation social distractors
Lin et al. (2022) [36]	PMDD = 63<br>NCF = 53	Executive function (Simon Task)<br>Attention (Attention and Performance Self-Assessment scale)<br>Fatigue (Fatigue Severity Scale)<br>Insomnia (Pittsburgh Insomnia Rating Scale)<br>Depression (The Center for Epidemiological Studies' Depression Scale)<br>Emotion regulation (Emotion Regulation Questionnaire)	Self-report tracking [b]	↓Cognitive reappraisal of emotions in PMDD
Lin et al. (2024) [37]	PMDD = 58<br>No PMDD = 50	ADHD symptoms (psychiatric assessment)<br>Attention (Attention and Performance Self-Assessment scale)	Self-report tracking [c]<br>Ovulation test	Diagnostic criteria for ADHD were met significantly more often in PMDD group<br>↓Attention in PMDD

		Impulsivity (Dickman Impulsivity Inventory)		
Pilarczyk et al. (2019) [27]	NCF = 20	Attention allocation to different stimuli (eye-tracking)	Saliva samples<br>Ovulation test	Faster RT, attention was allocated earlier to key regions of presented stimuli<br>P4 levels did not correlate significantly with any measure of visual attention
Pletzer et al. (2017) [33]	Males = 35<br>NCF = 32	Selective, divided and sustained attention (d2-R, FAIR-2, sustained attention test from the Wiener Test system)	Backwards counting [c]<br>Ovulation test	↓Selective/divided attention, ↓Sustained attention in NCF
Pletzer et al. (2019) [52]	NCF = 36	Spatial navigation (landmark test)<br>Verbal fluency (verbal fluency test)	Saliva samples<br>Ovulation test	No significant cognitive performance differences along the menstrual cycle<br>P4 boosts fronto-striatal activation
Roberts et al. (2018) [53]	NCF = 32	ADHD symptoms (Current ADHD Symptoms Scale: Self-report)<br>Trait impulsivity (UPPS-P Trait Impulsivity Scale)	Saliva samples (daily)	Decreased E2 + increased P4: ↑ADHD symptoms, especially with high trait impulsivity
Tuslyan et al. (2023a) [47]	NCF = 40	WM (dual-task n-back test)	Self-report tracking [b]<br>Backwards counting [b]	↑WM
Tuslyan et al. (2023b) [48]	NCF = 40	WM (dual-task n-back test)	Self-report tracking [b]<br>Backwards counting [b]	↑Target detection, performance remained stable<br>No significant differences were found across 3 menstrual cycles
Wang & Chen (2020) [31]	NCF = 26	Attention (Attention network test)<br>Emotional information processing (Emotional face flanker task)	Self-report tracking [c]<br>Saliva samples	Slower RT, ↑Accuracy<br>RT to sad faces correlated positively with P4 levels
Wang et al. (2025) [40]	Mid-luteal = 25	Attention, Executive function (face-gender Stroop task)<br>Resting-state fMRI<br>Task-based fMRI	Self-report tracking [c]<br>Saliva samples	↑Accuracy for female face stimuli only<br>P4 was positively correlated to differences in RT to female and male faces, and the nodal efficiency of inferior frontal gyrus in the resting-state executive control network
Xu et al. (2022) [32]	NCF = 79	Cognitive flexibility (Task-switching paradigm)	Backwards counting [b]	↑Sensitivity on divided attention task, ↑Cognitive flexibility

		Divided attention (Audiovisual cross-modal monitoring task)<br>Inhibition (spatial Stroop task)<br>Working memory (Multiple change detection paradigm)		
Zhuang et al. (2020) [54]	NCF = 16	Impulsivity (Monetary delay discounting task)<br>Resting-state fMRI<br>Task-based fMRI	Backwards counting [b]	↑ DlPFC activity in rest, which was sensitive to E2 levels
**Pre-menstrual**				
De Jong et al. (2023) [50]	NCF with ADHD and co-occurring conditions = 9	ADHD symptoms and pharmacotherapy (Community case study)	Self-report tracking [b]	↑ADHD and depressive symptoms, ↓Effectiveness medication<br>Increasing the stimulant dosage during the premenstrual week improved ADHD and mood symptoms with minimal adverse events
Eggert et al. (2017) [41]	PMS = 55<br>Non-PMS = 55	Attention, Executive Function (Emotional Stroop task)	Self-report tracking [b]	↑Emotional Stroop effect in PMS women
Lin et al. (2022) [36]	PMDD = 63<br>NCF = 53	Executive function (Simon Task)<br>Attention (Attention and Performance Self-Assessment scale)<br>Fatigue (Fatigue Severity Scale)<br>Insomnia (Pittsburgh Insomnia Rating Scale)<br>Depression (The Center for Epidemiological Studies' Depression Scale)<br>Emotion regulation (Emotion Regulation Questionnaire)	Self-report tracking [b]	↓Executive function, ↓Attention, ↓Cognitive reappraisal of emotions, ↑Insomnia, ↑Fatigue in PMDD<br>Inattention was the most associated factor of PMDD functional impairment
Lin et al. (2024) [37]	PMDD = 58<br>No PMDD = 50	ADHD symptoms (psychiatric assessment)<br>Attention (Attention and Performance Self-Assessment scale)	Self-report tracking [c]<br>Ovulation test	Diagnostic criteria for ADHD were met significantly more often in PMDD group<br>↓Attention, ↑Impulsivity in PMDD

		Impulsivity (Dickman Impulsivity Inventory)		
Yen et al. (2023) [38]	PMDD = 63<br>No PMDD = 53	Inhibition (Go/no-go task)<br>Attention (Go trials in Go/no-go task)<br>Impulsivity (Dickman's Impulsivity Inventory)	Self-report tracking [b]	↓Inhibition, ↓Attention, ↑Impulsivity in PMDD
**Full menstrual cycle**				
Louis et al. (2023) [45]	Met/Met [d]<br>NCF = 33<br>Val/Val [d]<br>NCF = 41	WM (n-back test)	Saliva samples (daily)	Val/Val**: when ↑E2, ↑WM (within-person)<br>Met/Met**: when ↑E2, ↓WM (within-person)<br>Within-person E2 levels were negatively correlated to RT
Roberts et al. (2018) [53]	NCF = 32	ADHD symptoms (Current ADHD Symptoms Scale: Self-report)<br>Trait impulsivity (Urgency, Premeditation, Perseverance, Sensation Seeking-Positive Urgency Trait Impulsivity Scale)	Saliva samples (daily)	ADHD symptoms were more state-like than trait-like<br>↑Impulsive traits, significant interactive effects of E2 and P4 on both inattention and hyperactivity

a. Studies are listed under multiple phases if they reported significant results in multiple menstrual phases. For full study details, see main Table 1.
b. Self-reported menstrual cycle phase; no hormonal verification conducted.
c. Self-reported menstrual cycle phase combined with hormonal verification method.
d. Val/Val and Met/Met are genotypes that have two copies of the valine or methionine variant of the COMT-gene.

Abbreviations: E2 = oestrogen, P4 = progesterone, NCF = naturally cycling females, OCF = females on oral contraceptives, RT = reaction time, WM = working memory, DA = dopamine, IUD = intrauterine device, ADHD = attention deficit hyperactivity disorder, PMDD = pre-menstrual dysphoric disorder, PMS = pre-menstrual syndrome, dlPFC = dorso-lateral prefrontal cortex.
